# The pattern and predictors of mortality of HIV/AIDS patients with neurologic manifestation in Ethiopia: a retrospective study

**DOI:** 10.1186/1742-6405-9-11

**Published:** 2012-04-10

**Authors:** Tesfaye Berhe, Yilma Melkamu, Amanuel Amare

**Affiliations:** 1 Department of Neurology, Addis Ababa University, Addis Ababa, Ethiopia; 2 School of Public Health, Addis Ababa University, Addis Ababa, Ethiopia

**Keywords:** Africa, Antiretroviral therapy, Cryptococcus, Opportunistic infection, Toxoplasmosis, Tuberculosis

## Abstract

**Background:**

Even though the prevalence of HIV infection among the adult population in Ethiopia was estimated to be 2.2% in 2008, the studies on the pattern of neurological manifestations are rare. The aim of this retrospective study was to assess the pattern and predictors of mortality of HIV/AIDS patients with neurologic manifestations.

**Methods:**

Medical records of 347 patients (age ≥13 years) admitted to Tikur Anbesa Hospital from September 2002 to August 2009 were reviewed and demographic and clinical data were collected.

**Results:**

Data from 347 patients were analysed. The mean age was 34.6 years. The diagnosis of HIV was made before current admission in 33.7% and 15.6% were on antiretroviral therapy (ART). Causes of neurological manifestation were: cerebral toxoplasmosis (36.6%), tuberculous meningitis (22.5%), cryptococcal meningitis (22.2%) and bacterial meningitis (6.9%). HIV-encephalopathy, primary central nervous system (CNS) lymphoma and progressive multifocal leukoencephalopathy were rare in our patients. CD4 count was done in 64.6% and 89.7% had count below 200/mm3[mean = 95.8, median = 57] and 95.7% were stage IV. Neuroimaging was done in 38% and 56.8% had mass lesion. The overall mortality was 45% and the case-fatality rates were: tuberculous meningitis (53.8%), cryptococcal meningitis (48.1%), cerebral toxoplasmosiss (44.1%) and bacterial meningitis (33.3%). Change in sensorium and seizure were predictors of mortality.

**Conclusions:**

CNS opportunistic infections were the major causes of neurological manifestations of HIV/AIDS and were associated with high mortality and morbidity. Almost all patients had advanced HIV disease at presentation. Early diagnosis of HIV, prophylaxis and treatment of opportunistic infections, timely ART, and improving laboratory services are recommended. Mortality was related to change in sensorium and seizure.

## Introduction

Symptomatic neurologic dysfunction develops in more than 50% of individuals infected with human immunodeficiency virus (HIV) [1] and about 10% experience neurologic symptoms as the initial manifestation of acquired immunodeficiency syndrome (AIDS) [2]. Neurologic disorders associated with HIV infection include central nervous system (CNS) infections, neoplasms, vascular complications, peripheral neuropathies and myopathies [3]. Neurologic dysfunction is an important cause or a strong marker of poor prognosis in late HIV infection [4].

Studies done in developing countries showed that the major causes of neurologic disorders in HIV/AIDS patients are CNS opportunistic infections [5-12]. In Ethiopia, the adult prevalence of HIV infection was estimated to be 2.2% in 2008 [13] but data on the pattern of neurological complications of HIV/AIDS are rare. This study was designed to assess the clinical presentation, cause, treatment, outcome and predictors of mortality in HIV/AIDS patients with neurological complications who were admitted to Tikur Anbesa Hospital (TAH), the main teaching hospital of Addis Ababa University.

## Patients and methods

In this retrospective study, HIV infected patients aged ≥ 13 years who were admitted with neurological manifestations of HIV/AIDS to TAH from September 2002 to August 2009 were included. Medical records of patients were retrieved and data sheets were used to collect the following data: age, sex, address, occupation, marital status, presenting symptoms and signs, investigations, treatment, duration of hospital stay, treatment outcome at hospital discharge.

Confidentiality was assured by giving each patient a unique number. The study was started after getting ethical approval from the appropriate body of the medical faculty of the university. Patients excluded from the study were: patients age < 13 years, patients who were not admitted, who had wrong diagnosis, or there were insufficient data for diagnosis.

Clinical staging of HIV/AIDS was made based on World Health Organization (WHO) staging system for HIV infection and disease [14]. The most recent CD4 count was taken into consideration. Poor outcome at hospital discharge was defined as patients who remained in the same clinical condition or deteriorated without death. Improved patients were patients who showed partial or complete recovery. The presumptive diagnosis of the neurological manifestations of HIV/AIDS was made as follows:

### Cerebral toxoplasmosis

Presumed diagnosis based on some or all of the following: i) headache, fever, change in sensorium ii) focal neurologic deficit iii) seropositivity for anti-toxoplasma immunoglobulin G(IgG), iv) ring enhancing brain lesion(s) on computerized tomography(CT)/magnetic resonance imaging(MRI), v) low CD4 count(< 200/mm3), and vi) response to toxoplasma treatment.

### Tuberculous meningitis

Based on some or all of the following: i) headache, fever, neck rigidity, change in mentation ii) cerebrospinal fluid (CSF) lymphocytosis iii) Acid-fast bacilli(AFB) on CSF smear iv) evidence of active tuberculosis in other part of the body (milliary tuberculosis, pulmonary tuberculosis etc.) v) CT/MRI showing meningeal enhancement vi) response to tuberculosis treatment.

### Tuberculoma

i) headache, change in sensorium ii) focal deficit iii) CT/MRI contrast enhancing lesion, and iv) response to tuberculosis treatment.

### Cyptococcal meningitis

CSF India ink positivity.

### Bacterial meningitis

i) acute onset headache, fever, change in mentation, neck rigidity ii) CSF polymorph predominant pleocytosis iii) CSF culture and Gram stain showing bacteria iv) response to antibiotics.

### Neurosyphilis

CSF Venereal Disease Research Laboratory (VDRL) reactive.

### Stroke

Sudden onset of neurologic deficit which persisted for > 24 h of presumed vascular origin.

### Guillian-Barre syndrome (GBS)/chronic inflammatory demyelinating polyneuropathy(CIDP)

Progressive weakness, paresthesias, areflexia, high CSF protein with pleocytosis, evidence of demyelination on nerve conduction studies.

### Primary CNS lymphoma

i) headache, change in sensorium, focal neurologic deficit ii) low CD4 count (< 200/mm3) iii) CT/MRI enhancing mass lesion iv) no response to toxoplasmosis and tuberculosis treatment

### Progressive multifocal leukoencephalopathy (PML)

i) multifocal progressive focal deficits, ii) CT/MRI multiple non-enhancing white matter lesions without edema or mass effect.

### Neurocyticercosis

multiple cerebral calcified lesions on CT scan.

### HIV encephalopathy

i) cognitive and motor abnormalities ii) CT/MRI showing brain atrophy iii) other opportunistic infections ruled out.

### Pseudotumor cerebri

i) clinical evidence of increased intracranial pressure ii) normal CT/MRI

The data was analysed using SPSS 13.0 for Windows (SPSS, Chicago IL, USA). Logistic regression was used to assess each possible prognostic factor. Odds ratios and significant levels were calculated along with 95% confidence interval. A p-value of less than 0.05 was considered significant. A multivariate logistic regression analysis was performed to determine which prognostic factors, when considered together, were the best predictors of hospital death.

## Results

Data from 347 patients were analysed. The mean age was 34.6 years (range 14-65) and 50.7% were males. Their marital status was married (52.2%), single (31.4%), widowed (7.8%), divorced (6.3%) and unknown (2.3%). The occupation was known for 305(87.9%) patients: housewives 86(24.8%), office employees 74(21.3%), car driver 36(10.4%), unemployed 31(8.9%), merchant 28(8.1%), daily laborer 21(6.1%), student 5(1.4%) and others 24 (6.9%).

Twenty-five percent (25%) came from outside Addis Ababa and the mean duration of symptoms was 30.6 days (range 1-240). The mean duration of symptoms for the major neurologic disorders identified was cerebral toxoplasmosis (25 days, median 14 days), tuberculous meningitis (34 days), cryptococcal meningitis (26 days) and bacterial meningitis (10 days). The diagnosis of HIV was made in 117 patients before current hospital admission and there was no statistically significant difference in case-fatality between patients who were diagnosed before and after admission (p = 0.315). The mean duration between diagnosis of HIV and current hospital admission was known for108 patients, and ranged between 1 and 120 months (mean 14.8 months). History of treatment with antiretroviral therapy (ART) was identified in 54(15.6%) patients with mean duration of treatment of 7.2 months (median 5 months, range1-29). ART was discontinued before hospital admission in 5 cases (They went to holy water). Co-trimoxazole prophylaxis was given to 15 cases before current admission and 2(13.3%) of these developed cerebral toxoplasmosis (p-value = 0.230).

The mean hospital stay was 21.9 days (range1-120). The mean hospital stay in days for improved and deceased patients was 26.4 (range 7-120) and 16.9 (range 1-91), respectively. The clinical manifestation of patients with neurological disorders is shown in Table 1 and the neurologic manifestation of HIV/AIDS in relation to outcome status is shown in Table 2. Herpes Zoster scar was identified in 67(19.3%) of cases. Co-morbidities identified at admission were pulmonary tuberculosis 18(5.2%) and pneumonia 18(5.2%). Some patients (n = 7, 2%) had more than one neurologic complications of HIV/AIDS: cryptococcal meningitis and neurosyphilis (n = 3), cryptococcal meningitis and GBS (n = 1), neurosyphilis and GBS (n = 1), tuberculous meningitis and tuberculoma (n = 1), cerebral toxoplasmosis and neurosyphilis (n = 1). The Glasgow coma scale (GCS) was recorded in 85(24.5%) of cases and 35(41.2%) had score of ≤ 8. The Case-fatality of patients with GCS score of ≤ 8 and > 8 was not different (p-value = 0.936). The WHO clinical staging of HIV/AIDS patients showed that majority (n = 332, 95.7%) were stage IV and the remaining were stage III (n = 15, 4.3%) cases.

**Table 1:** Clinical presentations of HIV/AIDS patients with neurological disorders

Sign/symptom	Cerebral toxoplasmosis(127)	Tuberculos meningitis(78)	Cryptococcal meningitis(77)	Bacterial meningitis(24)
Headache(212)	60(47.2%)	51(65.4%)	71(92.2%)	16(66.7%)

Altered sensorium(187)	74(58.3%)	57(73.1%)	28(36.4%)	18(75%)

Meningeal sign(136)	17(13.4%)	45(57.7%)	49(63.6%)	19(79.2)

Seizure(119)	54(42.5%)	27(34.6%)	21(27.3%)	6(25%)

Focal deficit(117)	83(65.4%)	9(11.5%)	5(6.5%)	1(4.2%)

Fever(108)	25(19.7%)	31(39.7%)	30(39%)	14(58.3%)

**Table 2:** Neurologic manifestations of HIV/AIDS in relation to outcome in 347 patients

Diagnosis	n(%)	Dead(156) n(%)	Poor outcome(21) n(%)	Improved(170) n(%)
cerebral toxoplasmosis	127(36.6)	56(44.1)	9(7.1)	62(48.8)

Tuberculosis meningitis	78(22.5)	42(53.8)	2(2.6)	34(43.6)

Cryptococcal meningitis	77(22.2)	37(48.1)	3(3.8)	37(48.1)

Bacterial meningitis	24(6.9)	8(33.3)	2(8.3)	14(58.3)

Tuberculoma	10(2.9)	4(40)	1(10)	5(50)

Neurosyphilis	9(2.6)	0	1(11.1)	8(88.9)

Stroke	8(2.3)	3(37.5)	0	5(62.5)

GBS	5(1.4)	1(20)	0	4(80)

Tuberculos spondylitis	5(1.4)	1(20)	2(40)	2(40)

Primary CNS lymphoma	3(0.9)	3(100)	0	0

CIDP	3(0.9)	0	0	3(100)

PML	2(0.6)	1(50)	0	1(50)

Others	3(0.9)	0	2(66.7)	1(33.3)

Note: *GBS *Guillain-Barre syndrome, *CNS *central nervous system, *CIDP *chronic inflammatory demyelinating polyneuropathy, *PML *progressive multifocal leukoencephalopathy, Others = pseudotumor cerebri (n = 1), neurocycticercosis (n = 1), HIV-encephalopathy (n = 1). Stroke: ischemic (n = 6), Hemorrhagic (n = 2).

### Investigations

CD4 count was done in 224(64.6%) cases and 201(89.7%) had count of below 200/mm3. The mean and median CD4 count per mm3 was 95.8 and 57, respectively. The mean CD4 count per mm3 for the major neurologic disorders identified was: cryptococcal meningitis = 55.5, cerebral toxoplasmosis = 66.3, tuberculous meningitis = 113.8 and bacterial meningitis = 204.4. Lumbar puncture was done in 226(65.1%) and the mean cell count was 467.3(range 1-8400). The mean differential count of neutrophil and lymphocyte was 39.2%(range 0-98) and 60.4%(range2-100), respectively. India ink was done in 216(62.2%) and was positive in 77(35.6%) cases. Out of 213(61.4%) patients tested for CSF VDRL, 9(4.2%) cases were reactive. Serology for toxoplasmosis (IgG) was done in 65(18.7%) and was positive in 44(67.7%) patients (titer was not done). CSF Gram stain and Acid fast stain showed organisms in 2 and 1 cases, respectively. Neuroimaging was done in 132(38%): CT-scan = 127, CT-scan and MRI = 2 and MRI = 3. The findings were: mass lesion (abscess and tumor) = 75(56.8%), normal = 30(22.7%), infarction = 13, meningeal enhancement = 5, brain atrophy = 5, non-enhancing white matter lesions = 2 and intracerebral hemorrhage = 2. Of the 75 patients with mass lesion on neuroimaging 60(80%) cases were diagnosed to have toxoplasmosis.

### Treatment

Toxoplasmosis was treated with sulfadoxine-pyrimethamine [pyrimethamine-sulfadiazine was not available]. Tuberculosis was treated with combination of Isoniazid, rifampin, pyrazinamide, ethambutol or streptomycine with pyridoxine. Cryptococcal meningitis was treated with oral fluconazole (n = 45) or Amphotericin B (n = 32). Seizure was treated with phenytoin in almost all patients. Dexamethasone intravenously was given (n = 182, 52.4%) and prednisolone used in 26(7.5%) cases. One or more intravenous antibiotics (ceftriaxone, crystalline penicillin, gentamycine etc.) was given to 212(61.1%) cases. ART was started after admission in 135(70.7% of cases discharged alive).

### Outcome

The case-fatality was 45%(n = 156) and the number of patients with poor outcome (discharged in the same clinical condition or deteriorated without death) was 6.1%(n = 21). In almost all patients, the immediate cause of death was attributable to the identified neurologic disorder(s).

Both univariate and multivariate logistic regression analysis (Table 3) showed that address from Addis Ababa, altered sensorium and seizure were predictors of mortality.

**Table 3:** Risk factor for case-fatality: logistic regression analysis

Factor		n	%	Unadjusted OR(95% CI)	Adjusted OR(95% CI)
Age(year)	< 40	113	44.3	1.00	1.00
	
	≥ 40	43	46.7	1.10(0.684-1.779)	1.033(0.529-2.018)

Sex	Male	77	43.8	1.00	1.00
	
	female	79	46.2	1.10(0.723-1.686)	1.033(0.566-1.887)

Address	Addis Ababa	133	50.8	1.00	1.00
	
	Outside Addis Ababa	23	27.1	0.360(0.210-0.615)	0.282(0.127-0.630)

History of ART treatment	Yes	21	38.9	1.00	1.00
	
	No	135	46.1	1.343(0.742-2.430)	1.1(0.506-2.393)

Cotrimoxazole (prophylaxis)	Yes	8	53.3	1.00	1.00
	
	No	148	44.6	0.704(0.249-1.986)	0.992(0.266-3.705)

Altered sensorium	Yes	99	52.9	1.00	1.00
	
	No	57	35.6	0.492(0.319-0.758)	0.514(0.288-0.916)

Seizure	Yes	65	54.6	1.00	1.00
	
	No	91	39.9	0.552(0.353-0.863)	0.519(0.288-0.935)

Clinical stage of HIV infection	Stage iii	3	20	1.00	1.00
	
	Stage iv	153	46.1	3.419(0.947-12.339)	2.069(0.370-11.562)

CD_4 _count(/mm^3^)	< 200	85	42.3	1.00	1.00
	
	≥ 200	6	26.1	0.482(0.182-1.273)	0.572(0.205-1.595)

*ART *antiretroviral treatment, *HIV *human immunodeficiency virus *CD_4 _*cluster of differentiation 4

## Discussion

The objectives of our study were to assess the clinical presentation, cause, treatment, outcome and predictors of mortality in patients with HIV/AIDS with neurologic complications admitted to the largest referral hospital in Ethiopia. Both sexes were equally affected unlike other studies [7,8,10,11] where males outnumbered females. Majority of our patients were young (mean age 34.6 years) which is similar to other studies [7-11]. There was delay at presentation (mean duration of symptoms prior to presentation was 30.6 days). The mean duration of symptoms prior to presentation for patients with cerebral toxoplasmosis (25 days) and cryptococcal meningitis (26 days) was similar to another study [2]. Majority (66.3%) of cases were known to have HIV infection after current hospital admission. Almost all patients had advanced HIV disease at presentation (4.3% stage III and 95.7% stage IV) which is consistent with another study [8].

The occurrence of seizure in cerebral toxoplasmosis in other studies [2,3] was 25% and 29%, respectively, which was lower compared to our finding. In another study [12], seizure occurred in 40% of patients with cerebral toxoplasmosis which was similar to our study. In a study [12] the occurrence of seizure in tuberculous meningitis (28%) and cryptococcal meningitis (50%) was lower and higher, respectively, compared with findings in our study. The common neurologic disorders identified in our cases were: cerebral toxoplasmosis (36.6%), tuberculous meningitis (22.5%) and cryptococcal meningitis (22.2%), and the frequencies were similar to other studies [5,6,11,12,15]. In contrary to our findings, tuberculous meningitis and bacterial meningitis were rare findings in patients with neurologic manifestations of HIV/AIDS in the United States [2]. In a study from India [12], cerebral toxoplasmosis was detected in 8.8%(5/57) which is very low compared to our finding. The number of patients with presumed diagnosis of primary CNS lymphoma was very low in our cases probably due to failure to do brain biopsy when empiric therapy for toxoplasmosis failed.

Stoke was identified in 2.3% of our patients which is low compared to a finding in another study [2] which was 12%. The low number of patients (2%) with multiple neurologic disorders compared to 13.5% in another study [2] probably shows the limited investigations available in our set up. Unlike other studies [2,16,17] HIV-encephalopathy was diagnosed in only one of our patients (0.28%).

Majority of our patients (89.7%) had a CD4 count below 200/mm3 which is consistent with another study [12]. Note that adult healthy Ethiopians have a mean absolute CD4 T-cell count of 775 cell/μL(range 366-1,235) in one study [18]. Neuroimaging studies in our cases showed focal lesions in 56.8% which is higher when compared to 25.5% obtained in another study [2]. The commonest (80%) cause of mass lesion on imaging studies in our patients was cerebral toxoplasmosis which is similar to other studies [1-3,5-7] but contrary to a study done in South Africa [9] where tuberculosis predominates as a cause of focal brain lesion(69%). In our study, normal neuroimaging finding and cerebral atrophy were detected in22.7% and 3.9%, respectively which is lower compared to another study [2]. These might be explained by the limited access to neuroimaging studies and the low incidence of HIV-encephalopathy, which is the main cause of brain atrophy in HIV/AIDS patients, in our cases. A study [19] on HIV-related neuropathology showed that the incidence of HIV-encephalopathy rises as patients survive longer after initiation of antiretroviral therapy and is the most common neurologic complications of HIV/AIDS in such set up [2]. In a study from Nigeria [10] which included ART naive patients only, HIV-encephalopathy was found in 12%(19/154) of cases which is higher when compared to our study. These show that history of treatment with ART alone does not explain the low incidence of HIV-encephalopathy in our patients. Our patients were highly selected group of patients with advanced disease stage in whom the neurologic symptoms might be overshadowed by overwhelming systemic illness. In addition, lack of adequate screening techniques and readily available imaging studies might contribute.

Amphotericin B was not readily available and treatment of cryptococcal meningitis was either Amphotericin B (n = 32) or oral fluconazole only (n = 45) [There was no significant difference in mortality, p-value = 0.260]. The limited availability of standard therapy might explain partly the higher case-fatality of cryptococcal meningitis (48.1%) in our cases compared to 32.4% in another study [2]. Sulfadiazine-pyrimethamine was not available and patients with cerebral toxoplasmosis were treated with pyrimethamin-sulfadoxin. This might partly explain the higher mortality in our set up (44.1%) compared to 30.2% in patients who were treated with standard treatment [2].

The overall hospital mortality was 45% which is high compared to other studies. In a study done in Nigeria [10] which included outpatients and inpatients, an overall 6-month mortality of 45% was found. In another study from India [11], a mortality of 34% was recorded. The in-hospital mortality of patients with neurologic manifestations of AIDS in Brazil [7] was 32%. The case-fatality for the major neurologic disorders in our study was: tuberculous meningitis (53.8%), cryptococcal meningitis (48.1%), cerebral toxoplasmosiss (44.1%) and bacterial meningitis (33.3%). In a study done in Cambodia [15], the case-fatality rates of tuberculous meningitis and cryptococcal meningitis was similar to our study which were 57% and 49%, respectively. In studies done in South Africa [20] and Nigeria [10] the case-fatality of HIV associated tuberculous meningitis was 71.8% and 77.5%, respectively, which is higher compared to our finding.

Both univariate and multivariate logistic regression analysis showed that address from Addis Ababa, altered sensorium and seizure were predictors of mortality. The unexpected relatively lower mortality of patients from outside the city of Addis Ababa might be partly explained by selection bias of cases who were relatively stable and able to travel to the city for better health care services. History of ART at admission was not found to improve mortality significantly.

This unexpected finding might be due to the relatively short period of treatment (mean 7 months, median 5 months), noncompliance (5 patients discontinued ART and of these 3 died) and only 15.6% of our cases were on ART. In patients with history of ART the common neurologic complications identified were: cerebral toxoplasmosis (n = 13), cyptococcal meningitis (n = 12), tuberculous meningitis (n = 10), bacterial meningitis (n = 9), stroke (n = 4) and others (n = 10). Further study is recommended to identify other possible reasons for the failure of ART in our patients.

There were several limitations to this study. Patients who were treated as outpatient only were not included and therefore the whole spectrum of neurologic manifestation might not be addressed. The absence of certain neurologic disorders in our study (myelopathy, peripheral neuropathy other than inflammatory demyelinating polyneurpathy, myopathy, etc.) might be partly explained by inclusion of inpatients only. Our hospital is a tertiary hospital and the chance of admitting selectively critically sick patients is high due to referral from other hospitals. The retrospective nature of the study might have caused unrecoverable missing data in some cases. There was no autopsy, biopsy, viral load and polymerase chain reaction done. CSF glucose and protein determination was done in minority of patients.

To improve care of patients with neurologic manifestations of HIV/AIDS, early diagnosis of HIV infection, patient education, starting prophylaxis when needed (e.g. INH and cotrimoxazole prophylaxis, BCG vaccination to decrease incidence of tuberculous meningitis), starting ART timely, making standard therapy for opportunistic infections readily available(e.g. sulfadiazine-pyremethamin with leucovorin and Amphotericin B), improving availability and quality of laboratory investigations and neuroimaging studies are very important.

In conclusion, most of our patients with neurologic manifestations of HIV/AIDS had advanced disease at presentations which was evidenced by: almost all were in stage IV, majority had low CD4 count and most had opportunistic CNS infections as the leading etiologies. Majority of patients were diagnosed to have HIV infection at admission and only a few patients were on ART and cotrimoxazole prophylaxis. Etiological diagnosis was difficult in some patients because of limited availability of laboratory services and lack of facilities for neuroimaging studies. Seizure and change in sensorium at presentation were independent predictors of mortality. To improve the high mortality and morbidity observed in our study, prevention of HIV infection, and when it occurs early diagnosis and appropriate prophylaxis and treatment of opportunistic infections and timely ART are recommended.

## Competing interests

The authors declare that they have no competing interests.

## Authors' contributions

AA wrote the first draft of the paper and all authors contributed comments and suggestions to various draft versions. TB participated in study design and data collection. YM participated in study design and coordinated the study. All authors have seen and approved the final manuscript.
